# European Registry on *Helicobacter pylori* Management (Hp-EuReg): Most relevant results for clinical practice

**DOI:** 10.3389/fgstr.2022.965982

**Published:** 2022-08-17

**Authors:** Olga P. Nyssen, Leticia Moreira, Natalia García-Morales, Anna Cano-Català, Ignasi Puig, Francis Mégraud, Colm O’Morain, Javier P. Gisbert

**Affiliations:** ^1^ Hospital Universitario de La Princesa, Instituto de Investigación Sanitaria Princesa (IIS-Princesa), Universidad Autónoma de Madrid (UAM), and Centro de Investigación Biomédica en Red de Enfermedades Hepáticas y Digestivas (CIBERehd), Madrid, Spain; ^2^ Hospital Clínic de Barcelona, Centro de Investigación Biomédica en Red en Enfermedades Hepáticas y Digestivas (CIBERehd), IDIBAPS (Institut d’Investigacions Biomèdiques August Pi i Sunyer), University of Barcelona, Barcelona, Spain; ^3^ Complexo Hospitalario Universitario de Vigo (CHUVI) and Galicia Sur Health Research Institute (IIS Galicia Sur), SERGAS-UVIGO, Vigo, Spain; ^4^ Althaia Xarxa Assistencial Universitària de Manresa, Universitat de Vic-Universitat Central de Catalunya (UVicUCC), Manresa, Spain; ^5^ INSERM U1312, Université de Bordeaux, Bordeaux, France; ^6^ Faculty of Health Sciences, Trinity College Dublin, Dublin, Ireland

**Keywords:** eradication, *Helicobacter pylori*, Hp-EuReg, registry, rescue, review, treatment

## Abstract

**Background:**

The ideal treatment approach for *H. pylori* infection has not yet been defined; therefore, the most effective management strategies for adult patients need to be identified to ensure clinical practice is aligned with the best standard of care. Our aim was to perform a review of research studies from the European Registry on *H. pylori* management (Hp-EuReg) by synthesizing the most clinically relevant information from each published manuscript.

**Methods:**

All research studies published between 2013 and 2022, evaluating any information related to *H. pylori* infection management within the Hp-EuReg, a long-term registry of routine clinical practice by gastroenterologists in Europe, were included in the review.

**Results:**

Overall, 26 studies have been published to date, where 12 evaluated the overall European data and the remaining were performed locally among the 28 participating countries. Eighteen studies evaluated the effectiveness of first- and/or second-line treatment, where one focused on penicillin allergic patients, six focused on specific treatment schemes, one evaluated the role of statins as a concomitant drug when combined with the eradication therapy, one assessed the adverse event profile of treatments, one evaluated the bacterial antibiotic resistance trends, and a last one reported on the common mistakes in routine clinical practice of European gastroenterologists.

**Conclusion:**

The Hp-EuReg had a major influence on the routine clinical practice of European gastroenterologists, improving *H. pylori* eradication treatment success, allowing to make recommendations in line with the current consensus guidelines and potentially serving as a model for other diseases.

## Introduction

*Helicobacter pylori* (*H. pylori*), a Gram-negative, flagellated, spiral bacterium, affects half of the population worldwide, that is, over 4.4 billion humans (1). The infection causes relevant diseases, such as gastritis, peptic ulcer disease, gastric mucosa-associated lymphoid tissue lymphoma, and gastric adenocarcinoma (2).

*H. pylori* eradication treatments aim to improve gastric mucosal inflammation, avoid progression of histologic damage, prevent ulcer recurrence, and reduce the incidence of gastric cancer and subsequently the incidence of deaths (3, 4).

Currently, the arbitrary—but reasonable—threshold for acceptance of a chosen treatment to cure any bacterial infection is settled to ≥90% (5–7). However, there are several issues associated with *H. pylori* eradication treatment that clinicians need to face in their routine clinical practice (8, 9).

Firstly, various treatment regimens are currently used worldwide (10), and the standard/recommended treatment varies with region and country, and so, nowadays, there is not a universally accepted regimen. Moreover, the *H. pylori* antimicrobial resistance is increasing worldwide due to indiscriminate antibiotic use (11). Indeed, determinants refer to drug availability and antibiotic susceptibility.

On the other hand, evidence derived from clinical trials may not be extrapolated to clinical practice, in which there are no restrictive inclusion criteria, and where available care time per patient and patient follow-up are more limited (12). Additionally, long-term studies should be performed; that is, data are collected from a population over time to look for trends and changes, in order to evaluate treatment management strategies and health research outcomes to provide real-time data from the routine clinical practice (local, regional, and global) (13). Moreover, these data collected at a population level can be used to generate hypothesis for further research, apart from generating data on causal relationships. Furthermore, it has been likewise highlighted the importance and strengths of the networked clinical collaboration speeding up the acquisition of results in one hand, increasing the intellectual mass of the project, gaining higher trust for participating investigators, and ultimately producing clinically relevant publications (14).

In this context, the “European Registry on *Helicobacter pylori* management” (Hp-EuReg) meets all aforementioned criteria, bringing together information on the real clinical practice in Europe (15), including currently over 50,000 patients from 28 countries with different bacterial resistance patterns and treatment accessibility. For these reasons, our aim was to perform a review of research studies from the Hp-EuReg by synthesizing the most clinically relevant information and messages from each published manuscript to date, in order to provide gastroenterologists with a guided overview of the current situation regarding *H. pylori* management as well as supplying an epidemiological model to monitor and supervise any infectious disease.

## European Registry on *H. pylori* management

The Hp-EuReg has been an ongoing international multicenter prospective non-interventional registry recording information of *H. pylori* infection management since 2013.

The project is conducted in accordance with the 1975 Declaration of Helsinki guidelines and was approved in 2012 by the Ethics Committee of La Princesa University Hospital (Madrid, Spain), the latter acting as reference Institutional Review Board. The Hp-EuReg was classified by the Spanish Drug and Health Product Agency, and was registered at ClinicalTrials.gov (NCT02328131).

A Scientific Committee acts as a steering group supervising all aspects of the project, paying special attention to the inclusion of data, analyses, and manuscript publications. In the published protocol (15) there is detailed information regarding the criteria for country selection, national coordinators, gastroenterologists recruiting investigators, and a list of variables and outcomes. Currently, 28 European countries with over 200 recruiters are participating. Each country is led by a national coordinator who ensures the active participation of the recruiting local investigators, promotes the study to maintain and boost recruitment, and is both the contact person and *liaison* with the Scientific Committee.

The list of countries and their corresponding national coordinators can be found in **Table 1**. The recruitment is heterogeneous, and 90% of the registry data are covered by the following countries (with highest to lowest participation): Spain, Russia, Italy, Slovenia, Lithuania, Azerbaijan, Norway, Latvia, Ukraine, and Greece.

**Table 1:** Current Hp-EuReg participating countries and their national coordinators.

Country	National Coordinator
**Azerbaijan**	Dr. Gulustan Babayeva
**Belgium**	Dr. Vincent Lamy
**Bulgaria**	Dr. Lyudmila Boyanova
**Croatia**	Dr. Ante Tonkić
**Czech Republic**	Dr. Lumír Kunovský
**France**	Dr. Tamara Matysiak-Budnik
**Germany**	Dr. Marino Venerito
**Greece**	Dr. Theodore Rokkas
**Hungary**	Dr. Gyorgy M Buzas
**Ireland**	Dr. Sinead Smith
**Israel**	Dr. Doron Boltin
**Italy**	Dr. Antonio Gasbarrini
**Latvia**	Dr. Marcis Leja
**Lithuania**	Dr. Juozas Kupčinskas
**Netherlands**	Dr. Lisette Capelle
**Norway**	Dr. Frode Lerang
**Poland**	Dr. Wojciech Marlicz
**Portugal**	Dr. Ricardo Marcos Pinto
**Romania**	Dr. Daniela Dobru
**Russia**	Dr. Dmitry Bordin
**Serbia**	Dr. Vladimir Milivojevic
**Slovenia**	Dr. Bojan Tepes
**Spain**	Dr. Natalia García-Morales
**Sweden**	Dr. Per Hellström
**Switzerland**	Dr. Michael Doulberis
**Turkey**	Dr. Halis Simsek
**Ukraine**	Dr. Oleksiy Gridnyev
**United Kingdom**	Dr. Perminder Phull

Data are recorded in an Electronic Case Report Form (e-CRF), collected and managed using the web-based application designed to support data capture for research studies, REDCap (Research Electronic Data Capture), hosted at “Asociación Española de Gastroenterología” (AEG; www.aegastro.es), a non-profit Scientific and Medical Society focused on gastroenterology research. Data are systematically extracted and continuously checked to both explode and maintain the quality of the database.

**Figure 1:**
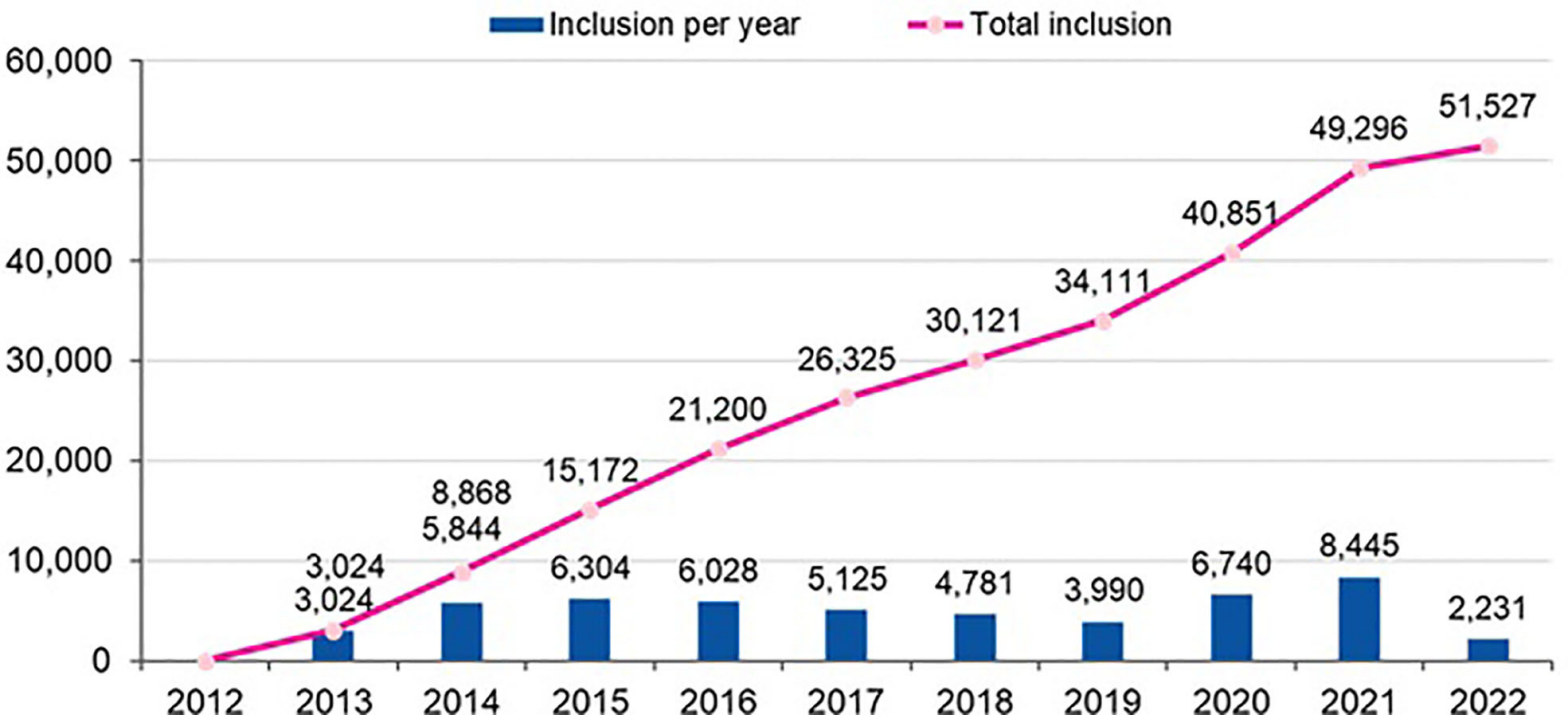
Hp-EuReg participation rate (number of patients per year; updated in April 2022).

The primary aim of the Hp-EuReg is to obtain a comprehensive database registering systematically a large and representative sample of routine clinical practice of European gastroenterologists in order to produce descriptive studies of the management of *H. pylori* infection. Currently, the Hp-EuReg has included over 50,000 patients with an inclusion rate of 4,000–6,000 patients per year, except for the year 2021, registering the highest participation to date, with more than 8,000 patients collected (**Figure 1**).

The secondary aims of the Hp-EuReg are as follows: to evaluate *H. pylori* infection consensus and clinical guidelines implementation in different countries; to perform studies focused on epidemiology, efficacy, and safety of the commonly used treatments to eradicate *H. pylori* in adults’ patients; to evaluate accessibility to healthcare technologies and drugs used in the management of *H. pylori* infection; and, ultimately, to allow the development of partial and specific analyses by the participating researchers.

All studies’ analyses on effectiveness are based on three different patient groups: (1) an intention-to-treat (ITT) analysis including all cases collected according to the time span of the given study, and allowing a minimum of a 6-month follow-up; in the ITT analysis, cases not returning to the clinic after treatment and where the confirmation of the eradication (i.e., success or failure) was not available were considered treatment failures; (2) a per-protocol (PP) analysis including patients with a confirmation of the eradication and those having taken at least 90% of the treatment drugs, as defined in the protocol; lastly, (3) a modified ITT (mITT) analysis, aiming to mimic the clinical practice outcomes and including all records with a confirmatory test after the eradication treatment, regardless of compliance. For the purpose of the current review, only the results on the mITT analysis will be used for the evidence synthesis.

Adverse events (AEs) and compliance are evaluated through patient questioning using both open-ended questions and a predefined questionnaire. Compliance is defined as having taken ≥90% of the prescribed drugs. In the present review, the AEs’ overall incidence and the compliance overall rate will be used to synthesize the information.

Regarding treatment duration, all studies use three categories: 7, 10, and 14 days, which are the most frequently used lengths; and the proton pump inhibitor (PPI) type and dosages used in combination with the antibiotics are also categorized using the omeprazole equivalent (OE) reference in milligrams per day, as elsewhere described (16, 17): low dose (4.5–27 mg of omeprazole equivalents given twice a day), standard dose (32–40 mg of omeprazole equivalents given twice a day), or high dose (54–128 mg of omeprazole equivalents given twice a day).

Most up-to-date and relevant information of the Hp-EuReg, such as the research team, the list of all publications derived from the study, the participating countries, and graphs summarizing the main first-line and rescue treatments’ prescriptions, is presented in the following website: http://www.hpeureg.com/.

## Relevant information for the clinician

In total, 26 studies were reviewed one-by-one in a narrative way and the main results and conclusions were synthesized in chronological order in a tabular summary (**Table 2**), including the following items: first author and publication date, objectives, main results, and conclusions.

**Table 2:** Tabular summary of studies included in this review.

First author, Year of publication	Objective	Main results and conclusions
Gisbert J. P., 2015 (18)	To evaluate the efficacy and tolerability of a second-line quadruple regimen containing levofloxacin and bismuth in patients whose previous *H. pylori* eradication treatment failed.	Overall, 200 patients from Spain and Italy were included consecutively.
		Previous failed therapy included: standard clarithromycin triple therapy (131 patients), sequential (32) and concomitant (37).
		PP and ITT eradication rates were 91.1% and 90%. Cure rates were similar regardless of previous (failed) treatment or country of origin.
		AEs were reported in 46% of patients, most commonly nausea (17%) and diarrhea (16%); 3% were intense but none was serious.
Molina-Infante J., 2015 (19)	To compare the efficacy and safety of optimized triple (OPT-TRI) and non-bismuth quadruple concomitant (OPT-CON) first-line therapies.	The OPT-CON therapy achieved significantly higher eradication rates in the PP (82.3%) vs. 93.8%, *p* < 0.001) and ITT analysis (81.3% vs. 90.4%, *p* < 0.001).
		Adverse events (97% mild/moderate) were significantly more common with OPT-CON therapy (39% vs. 47%, *p* = 0.016).
		Addition of metronidazole to an OPT-TRI therapy increased eradication rates by 10%, resulting in a higher rate of mild adverse events but without impairing compliance with therapy.
		Over 90% cure rates with an OPT-TRI were also observed in few centers as a consequence to either known clarithromycin susceptibility or empirically limited to settings where successful cure rates (>90%) were previously identified.
		In conclusion, an OPT-CON therapy might be preferable as empiric first-line therapy for *H. pylori* in areas with increasing clarithromycin resistance rates.
Bordin D., 2016 (20)	To assess the clinical practice of diagnosis and treatment in patients with *H. pylori* infection in Russia and to compare this practice with the international guidelines.	·The most common methods for the primary diagnosis of *H. pylori* infection were histology (40.3%), rapid urease test (35.7%), and serology (17.2%). The duration of *H. pylori* eradication therapy was 7, 10, and 14 days in 18.0, 49.3, and 25.1%, respectively.
		To monitor the effectiveness of treatment, the investigators used a histological examination (34%), a urea breath test (27.3%), *H. pylori* stool antigen (22.8%), and a rapid urease test (16.3%). A serological test was carried out in 2.5% of the cases.
		No monitoring was done in 13.5% of the patients. The average eradication efficiency was 82.6%. If the therapy was ineffective, 80% of physicians did not intend to prescribe a new cycle of treatment.
Bordin D., 2018 (21)	To evaluate the real clinical practice of diagnosis and treatment of *H. pylori* in Russia and its comparison with international recommendations.	The most common methods of primary diagnosis of *H. pylori* were histological (37.7%), rapid urease test (29.2%), and serology (29.7%). The duration of eradication therapy in 9.4% of cases was 7 days; in 65.3%, 10 days; and in 25.3%, 14 days.
		To control the effectiveness of treatment, *H. pylori* antigen in feces (31.3%), urea breath test (23.4%), and histological method (23.3%) were used. In 3.6% of cases, serology was used by mistake. In 17.3% of patients, control was not carried out.
		The effectiveness of triple therapy with a PPI, amoxicillin, and clarithromycin (PP) was 67.6% with a 7-day course, 81.1% with 10 days, and 86.7% with 14 days.
		Eradication rate of triple therapy with the addition of bismuth (PP) reached 90.6% in the group receiving the 10-day scheme and 93.6% in the group receiving the 14-day treatment.
		Significant deviations of clinical practice from expert recommendations, most pronounced at the stage of monitoring the effectiveness of therapy, were noted. The suboptimal efficacy of triple therapy was shown.
Tepes B., 2018 (22)	To analyze the data for *H. pylori* eradication treatments in Slovenia from 2013 to 2015.	Overall, 1,774 patients were analyzed: 1,519 patients in the PP set and 1,346 in the mITT.
		Eradication rate for first-line 7-day triple therapy with PPI, clarithromycin, and amoxicillin was 88.7% PP and 72.0% mITT; for PPI, clarithromycin, and metronidazole, it was 85.2% PP and 84.4% mITT.
		Second-line 14-day therapy PPI, amoxicillin, and levofloxacin had 92.3% eradication rate PP and 87.1% mITT.
		Ten- to 14-day bismuth quadruple therapy was the therapy in difficult-to-treat patients.
		Patients’ dropout was 11.4%. All patients that adhered to prescribed regimens were cured of their *H. pylori* infection.
Bordin D., 2019 (23)	To analyze the real clinical practice of diagnosis and treatment of *H. pylori* in Russia and its comparison with international recommendations	Invasive diagnostic methods prevail for the primary diagnosis of *H. pylori* [histology—20.3% (in 2013) and 43.9% (in 2018); rapid urease test—31.7% and 47.8%, respectively].
		The most popular mode of eradication therapy was a 10-day triple therapy (62.8%–76.2%), the effectiveness of which did not exceed 79% (PP).
		Invasive tests (histology) are the leading method for controlling the effectiveness of therapy; however, there is a tendency towards a wider use of non-invasive methods (*H. pylori* stool antigen—from 17% in 2013 to 29.3% in 2018 and urea breath test from 6.9% to 18.3%, respectively). Serological test to control the effectiveness of eradication is still used from 8.2% (2013) to 6.1% (2018).
		Eradication therapy was not performed in 28% of patients throughout the entire observation period.
		In Russia, despite approved domestic and international recommendations, deviations in clinical practice persist, both during eradication therapy and in monitoring the effectiveness of eradication therapy.
Buzas G., 2019 (24)	To assess the efficacy of different *H. pylori* eradication regimens in a single outpatient clinic of gastroenterology in Hungary.	As first-line treatment, the patients received either a 7-day triple regimen (PPI, amoxicillin, clarithromycin, or tinidazole), a 10-day modified sequential treatment (PPI, amoxicillin for 5 days, tinidazole, and levofloxacin for 5 days), a 10-day quadruple concomitant treatment (PPI, amoxicillin, tetracycline or doxycycline and metronidazole or tinidazole), or a bismuth-based quadruple treatment. Bismuth or non-bismuth-based quadruple or alternative regimens were given as second- or third-line treatment.
		The eradication rates on PP basis were as follows: 82.7% (first-line regimens), 85.2% (sequential treatment), 95.1% (concomitant treatment), and 82.6% (bismuth-based quadruple regimen). Second-line regimens achieved 65.2% and third-line therapy 54.5% cure rates, respectively.
		The first-line concomitant regimen was superior to triple and not significantly better than the sequential or bismuth-based treatment. Second- and third-line regimens achieved largely suboptimal results.
McNicholl AG, 2020 (25)	To assess the effectiveness and safety of the combination of bismuth and the standard, clarithromycin-containing triple therapy in the eradication of *H. pylori* infection.	Addition of bismuth to triple therapy achieved acceptable rates of. *H. pylori* eradication (approximately 90%).
		Treatments lasting 14 days and including a double dose of PPI (equivalent to 40 mg omeprazole, twice daily) increased efficacy.
		This treatment had an acceptable safety profile, comparable to that of other eradication treatments.
Nyssen O. P., 2020 (26)	To evaluate the efficacy and safety of first-line and rescue treatments in patients allergic to penicillin in Europe.	Overall, 1,084 patients allergic to penicillin from 27 countries were analyzed.
		The most frequently prescribed first-line treatments were as follows: triple therapy with clarithromycin and metronidazole (48%) and bismuth quadruple therapy with metronidazole and tetracycline (classic or Pylera^®^; 42%).
		In first-line treatment, the efficacy of triple therapy with clarithromycin and metronidazole was 69%, while bismuth quadruple therapy with metronidazole and tetracycline reached 91% (*p* < 0.001).
		In second-line treatment, after the failure of triple therapy with clarithromycin and metronidazole, two rescue options showed similar efficacy: bismuth quadruple therapy with metronidazole and tetracycline (78%) and triple therapy with clarithromycin and levofloxacin (71%) (*p* > 0.05).
		In third-line treatment, after the failure of triple with clarithromycin and metronidazole and triple with clarithromycin and levofloxacin, bismuth quadruple therapy with metronidazole and tetracycline was successful in 75% of cases.
Caldas M., 2020 (27)	To evaluate the effectiveness of first- and second-line *H. pylori* treatment in Spain, where the empirical prescription is recommended	In Spain, the standard triple therapy containing clarithromycin and amoxicillin shows an inadequate effectiveness; and therefore, its empirical use should be abandoned.
		The best effectiveness in first-line therapy was obtained with the bismuth single capsule prescribed for 10 days, and the concomitant and bismuth-clarithromycin quadruple therapies, both prescribed for 14 days.
		In second-line treatment, the best effectiveness was obtained with a 10-day bismuth single-capsule regimen, and also with 14-day quinolone-containing therapies, either with or without bismuth.
		A tendency over time towards the rise in the use of quadruple therapies, longer duration regimens, and higher doses of PPIs was observed, which is in line with current recommendations. These three factors, together with good compliance, seem to be, in general terms, the four main strategies to increase effectiveness.
Nyssen O.P., 2020 (28)	To evaluate the efficacy and safety of third-line treatments with bismuth, metronidazole, and either tetracycline or doxycycline.	Tetracycline-containing bismuth quadruple treatment (either with the traditional or with the three-in-one formulation) is an acceptable and safe third-line alternative after two previous *H. pylori* eradication failures with key antibiotics such as amoxicillin, clarithromycin, and levofloxacin.
		The use of doxycycline instead of tetracycline in the context of a bismuth quadruple therapy should not be recommended, as it seems to be less effective.
Abdulkhakov S. R., 2020 (29)	To assess the compliance of clinical practice in the management of patients with *H. pylori* infection in Kazan with clinical guidelines	Overall, 437 patients were analyzed up to 2019.
		The rapid urease test (44.2% of cases) and cytology/histology (60% of cases) were most often used for the initial diagnosis of *H. pylori* infection; however, non-invasive methods such as 13C-urea breath test (9.2%), serology (6.2%), and stool antigen test (2.3%) were less common. In 21.7% of patients, two methods of *H. pylori* detection were used for primary diagnosis.
		The control test to evaluate the effectiveness of eradication therapy at the recommended time point was performed in 46.2% of patients. 13C-urea breath test (31.7%), stool PCR/stool antigen test (28.7%), rapid urease test (22.3%), and cytology/histology (26.2% of cases) prevailed in the assessment of eradication rate.
		Standard triple therapy with PPI, clarithromycin, and amoxicillin was most commonly prescribed as first-line therapy (64.6% of cases). The duration of eradication therapy was 14 days in the majority of cases, with pantoprazole as the most common PPI in standard triple-therapy regimens (84.8%).
		The effectiveness of the 14-day standard triple therapy (mITT) was 87%.
		In conclusion, the results indicate a high frequency of non-invasive methods used for assessing the effectiveness of eradication therapy. The effectiveness of the most common 14-day standard triple first-line therapy in Kazan does not reach the recommended 90% eradication level. This could be explained by the high rate of pantoprazole use, which is not an optimal PPI in eradication therapy regimens.
Bordin D., 2020 (30)	To evaluate the effectiveness of the standard triple therapy (PPI, amoxicillin, and clarithromycin) and the standard triple therapy plus bismuth tripotassium dicitrate.	A total of 647 patients were collected, and 330 were administered either standard triple therapy or standard triple therapy plus bismuth.
		Invasive methods were most often used in the initial diagnosis of *H. pylori*: rapid urease test use decreased from 50% in 2013 to 31% in 2019. Serology was used in 27.9%. An increase in the use of the 13C-urea breath test from 13% in 2013 to 31% in 2019 was noted. The histological method (7.5%) and the stool antigen test (3.2%) were used less frequently.
		For eradication control, non-invasive methods were mostly used: 13C-urea breath test (82.7%) and the stool antigen test (14.4%).
		The effectiveness of standard triple therapy was 68% with a 7-day course, 79% with 10 days, and 70% with 14 days. The combination of bismuth and standard triple therapy eradicates *H. pylori* in 63%, 75%, and 89%, respectively.
		In summary, an improvement in the clinical practice of managing patients with *H. pylori* infections was noted. The standard triple therapy in combination with bismuth, prescribed for 14 days, was more effective.
Nyssen OP, 2021 (31)	To establish a large-scale, long-term prospective clinical practice study providing an overview of the current situation regarding *H. pylori* management in Europe.	Triple-therapy prescriptions (reporting cure rates of approximately 80%) have decreased, especially in those regions with high clarithromycin resistance.
		Over 90% eradication was only obtained with 10-day bismuth quadruple therapies or 14-day concomitant treatment.
		From 2013 to 2018, the observed shift to longer treatment duration, higher acid inhibition, and compliance provided an increase in the effectiveness.
Nyssen O. P., 2021 (32)	To evaluate the effectiveness and safety of the single‐capsule bismuth quadruple therapy.	The development of a three‐in‐one single‐capsule formulation has led to a resurgence in the use of bismuth quadruple therapy to treat *H. pylori* infection.
		In the largest study carried out to date, the effectiveness of the single capsule was optimal both as a first-line therapy and as a rescue therapy.
		Compliance was the factor most closely associated with treatment effectiveness.
		Single‐capsule bismuth quadruple therapy eradicates *H. pylori* in approximately 90% of patients in real‐world clinical practice, with a favorable safety profile.
Nyssen O. P., 2021 (33)	To evaluate the frequency of use, the effectiveness, and the safety of second-line empirical treatment in Europe.	Overall, 5,055 patients received empiric second-line treatment. Triple therapy with amoxicillin and levofloxacin was prescribed most commonly (33%).
		The overall effectiveness was 82% by mITT analysis, and 83% by PP.
		After the failure of first-line clarithromycin-containing treatment, optimal eradication (>90%) was obtained with moxifloxacin-containing triple therapy or levofloxacin-containing quadruple therapy (with bismuth).
		In patients receiving triple therapy containing levofloxacin or moxifloxacin, and levofloxacin-bismuth quadruple treatment, cure rates were optimized with 14-day regimens using high doses of PPIs.
		Bismuth quadruple single-capsule or levofloxacin-bismuth quadruple therapy produced reliable eradication rates regardless of PPI dose, duration of therapy, or previous first-line treatment.
		AEs were reported in 28%, and most (85%) were mild. However, 0.3% were serious and required hospitalization.
Nyssen O.P., 2021 (34)	To assess the frequency, type, intensity, and duration of AEs, and their impact on compliance, for the most frequently used treatments in Europe.	The different treatments prescribed to 22,492 patients caused at least 1 AE in 23% of the cases, with an average duration of 7 days.
		Classic bismuth quadruple therapy was the worst tolerated (37% of AEs).
		Most frequent AEs were as follows: taste disturbance (7%), diarrhea (7%), nausea (6%), and abdominal pain (3%). The majority of AEs were mild (57%), 6% were severe, and 0.08% were serious.
		Compliance was 97%, but 1.3% of patients discontinued treatment due to AEs.
		Longer treatment durations were significantly associated with a higher incidence of AEs in standard triple, concomitant, bismuth quadruple, and levofloxacin triple or quadruple therapies.
Caldas M., 2021 (35)	To evaluate if the concomitant use of statins prescribed for cardiovascular prevention and chronically used could modify the effectiveness rates of *H. pylori* eradication therapies.	Overall, 9,988 and 705 patients received empirical and culture-guided treatment, respectively.
		Statin use was associated with higher effectiveness in the empirical group, but no association was found with first-line treatment effectiveness (*n* = 7,738); as an exception, statin use was specifically associated with lower effectiveness of standard triple therapy.
		In the rescue therapy empirical group (*n* = 2,228), statins were associated with higher overall effectiveness; however, sub-analyses by treatment schemes only confirmed this association for the single-capsule bismuth quadruple therapy.
		No consistent association was found between statin use and *H. pylori* therapy effectiveness. Therefore, the addition of statins to the usual *H. pylori* treatment cannot be currently recommended to improve cure rates.
Bujanda L., 2021 (36)	To conduct a time-trend analysis of *H. pylori* resistance to antibiotics in Europe.	Overall, 2,852 (7%) were naïve cases included for analysis. The number of positive cultures decreased by 35% from the period 2013–2016 to 2017–2020.
		No antibiotic resistance was found in 48% of the naïve cases and the most frequent were against metronidazole (30%), clarithromycin (25%), and levofloxacin (20%), whereas resistances to tetracycline and amoxicillin were below 1%. Dual and triple resistances were found in 13% and 6% of the cases, respectively.
		A decrease (*p* < 0.001) in the metronidazole resistance rate was observed between the 2013–2016 (33%) and 2017–2020 (24%) periods.
Tepes B., 2021 (37)	To analyze the data for *H. pylori* eradication treatments in Slovenia from 2017 to 2019 after the third national recommendations were implemented.	Overall, 853 patients were evaluated.
		Effectiveness with first-line 14-day triple therapy with a PPI, clarithromycin, and amoxicillin was 93% by mITT (714 patients).
		In patients allergic to penicillin, first-line 14-day triple therapy with PPI, clarithromycin, and metronidazole achieved 83% effectiveness by mITT (35 patients).
		Second-line 14-day triple therapy with a PPI, amoxicillin, and levofloxacin achieved 89% mITT eradication rate (51 patients). Second-line therapy with the 10-day three-in-one single-capsule containing bismuth-tetracycline-metronidazole achieved optimal effectiveness (100% mITT) in 10 patients.
Jonaitis P., 2021 (38)	To evaluate the diagnostic methods and treatment of *H. pylori* infection as well as their adherence to Maastricht V/Florence consensus during the years 2013–2020 in Lithuania.	Triple therapy was used in 90% of the cases. In 91% of the first-line prescriptions, standard triple therapy was used. The most common second-line treatment was a combination of PPI, amoxicillin, and levofloxacin (47%).
		The overall effectiveness in 552 cases valid for analysis was 90% by mITT. In first-line treatment, the standard triple-therapy effectiveness was 90%, and second-line treatment with PPI, amoxicillin, and levofloxacin achieved 92% by mITT.
		Increasing overall *H. pylori* eradication rates were observed: from 72% in 2013 to more than 90% in 2018–2020, as well as a shift from 7- to 10- to 14-day treatment duration throughout 2013–2020.
Boltin D., 2021 (39)	To determine the effectiveness, compliance, and safety of first-line treatment for *H. pylori* in Israel.	In total, 242 patients were registered, including 121 (50%) who received first-line therapy; 41% of these individuals received clarithromycin-based triple therapy, and 58.9% received a four-drug regimen.
		The overall effectiveness of first-line therapy was 85% and 86% by mITT and PP analyses, respectively. The effectiveness of both sequential and concomitant therapies was 100%, while clarithromycin-based triple therapy achieved an eradication rate of 79%.
		Treatment eradication was higher among patients who received high-dose PPIs compared to those treated with low-dose PPIs (100% vs. 81.5% respectively, *p* < 0.01).
		No difference in treatment effectiveness was found between 7-, 10-, and 14-day treatment.
Nyssen O. P., 2022 (40)	To assess the effectiveness and safety of *H. pylori* regimens containing rifabutin in Europe.	Overall, 500 patients were treated with rifabutin, most of them as part of a triple therapy together with amoxicillin and a PPI, and in an additional 6% of the patients, bismuth was added to this triple regimen.
		Rifabutin was mainly used in second-line (32%), third-line (25%), and fourth-line (27%) regimens, achieving overall 78%, 80%, and 66% effectiveness by mITT, respectively.
		Compliance was 89%. AE incidence was 26% (most frequently nausea) and one serious adverse event (0.2%) was reported in one patient with leukopenia and thrombocytopenia with fever requiring hospitalization.
Nyssen O. P., 2022 (41)	To evaluate the common mistakes in the routine of the clinical practice in the eradication treatment of *H. pylori* in Europe.	In total, 26,340 patients were evaluated.
		The most common mistakes were as follows: (1) To use the standard triple therapy where it is ineffective (46%). (2) To prescribe eradication therapy for only 7 to 10 days (69%). (3) To use a low dose of PPIs (48%). (4) In patients allergic to penicillin, to prescribe always a triple therapy with clarithromycin and metronidazole (38%). (5) To repeat certain antibiotics after eradication failure (>15%). (6) Failing to consider the importance of compliance with treatment (2%). (7) Not to check the eradication success (6%).
		The time-trend analyses showed progressive greater compliance with current clinical guidelines.
Rokkas T., 2022 (42)	To evaluate treatment effectiveness in naïve patients in Greece.	Overall, 547 patients were treated with the following regimens (% overall use):
		From 2013 to 2015, triple with PPI, clarithromycin, and amoxicillin (7%); sequential with PPI, clarithromycin, amoxicillin, and metronidazole or tinidazole (12% each); and concomitant with PPI, clarithromycin, amoxicillin, and tinidazole (8%) were used. The respective mITT cure rates were 92%, 87%, 67%, and 91%.
		Since 2015, patients were also treated with concomitant with PPI, clarithromycin, amoxicillin, and metronidazole (38%) and hybrid with PPI, clarithromycin, amoxicillin, and metronidazole (20%) regimens, with respective mITT cure rates of 90% and 88%.
		Overall compliance was 99%. AEs were reported by 31% of the patients, dysgeusia being the most frequent (15%).
Fernandez-Salazar L., 2022 (43)	To evaluate the frequency of use, the effectiveness, the compliance, and the safety of the high-dose dual therapy (HDDT) regimen in the management of *H. pylori* infection in Europe	HDDT was prescribed to 60 patients: 19 cases were treatment-naïve and 41 were treated as rescue therapy. Overall mITT HDDT effectiveness was 51% with no statistical differences between treatment lines. Effectiveness decreased in those who had been previously treated with metronidazole, tetracycline, or rifabutin. The addition of bismuth to HDDT did not increase the cure rates. The incidence rate of at least one AE was 30% (diarrhea most commonly in 20%). No serious AEs were registered. In conclusion, HDDT (with or without bismuth) does not represent a good therapeutic option in Europe in any line of treatment.

AEs, adverse events; ITT, intention-to-treat; mITT, modified intention-to-treat; PP, per-protocol; PPI, proton pump inhibitor.

Additionally, 20 further manuscripts are currently on preparation (**Supplementary Table 1**).

In this section, the studies assessing global data from Europe will be presented first, followed by the local data (by country).

### European global data

#### European Registry on Helicobacter pylori management (Hp-EuReg): Patterns and trends in first-line empirical eradication prescription and outcomes of 5 years and 21,533 patients

Nowadays, the best therapeutical approach to eradicate *H. pylori* infection remains unclear, and thus, a continuous and systematic audit is essential to ensure and improve the best therapeutic options, according to different geographic settings and their drug accessibility.

The objective of this study was to evaluate the effectiveness and safety of first-line treatments across European regions and to perform both trend and geographical analysis (31). The data extraction covered a 5-year period, that is, from May 2013 to December 2017.

**Figure 2:**
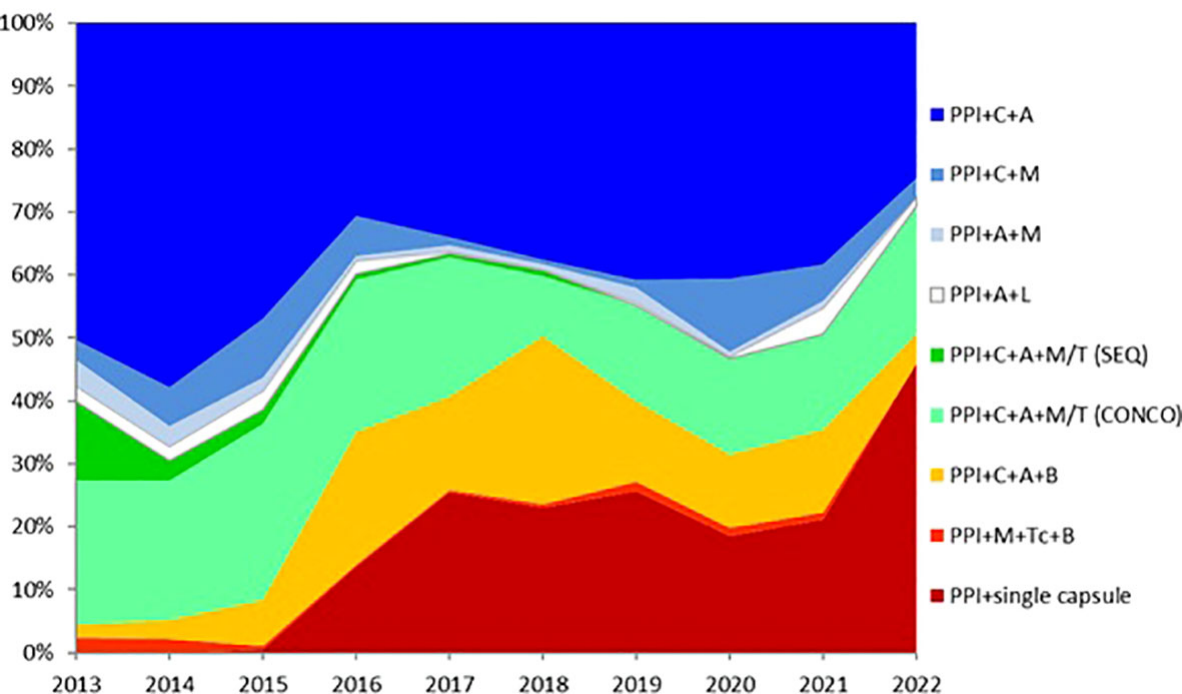
Time trends in the most common first-line treatment prescriptions in Europe (updated in April 2022). PPI—proton pump inhibitor; Seq—sequential; Conc—concomitant; C—clarithromycin; L—levofloxacin; M—metronidazole; T—tinidazole; A—amoxicillin; B—bismuth salts; Tc—tetracycline; single capsule—containing bismuth, tetracycline, and metronidazole.

**Figure 3:**
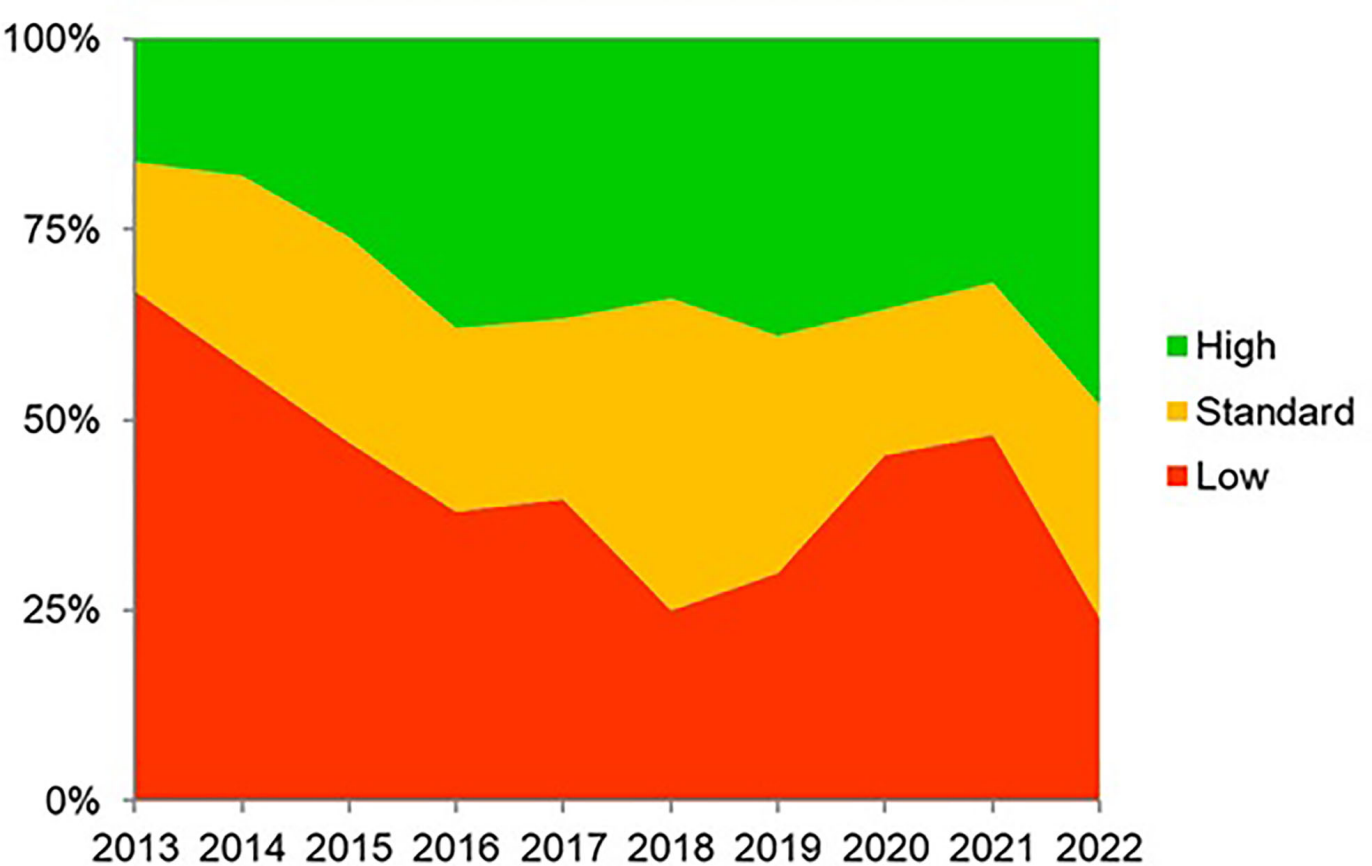
Trends (2013–2022) in the daily dose (low, standard, and high) of proton pump inhibitor prescriptions in Europe (updated in April 2022). Categories using the omeprazole equivalent (OE) reference in milligrams per day: low dose (4.5–27 mg of omeprazole equivalents given twice a day), standard dose (32–40 mg of omeprazole equivalents given twice a day), or high dose (54–128 mg of omeprazole equivalents given twice a day).

**Figure 4:**
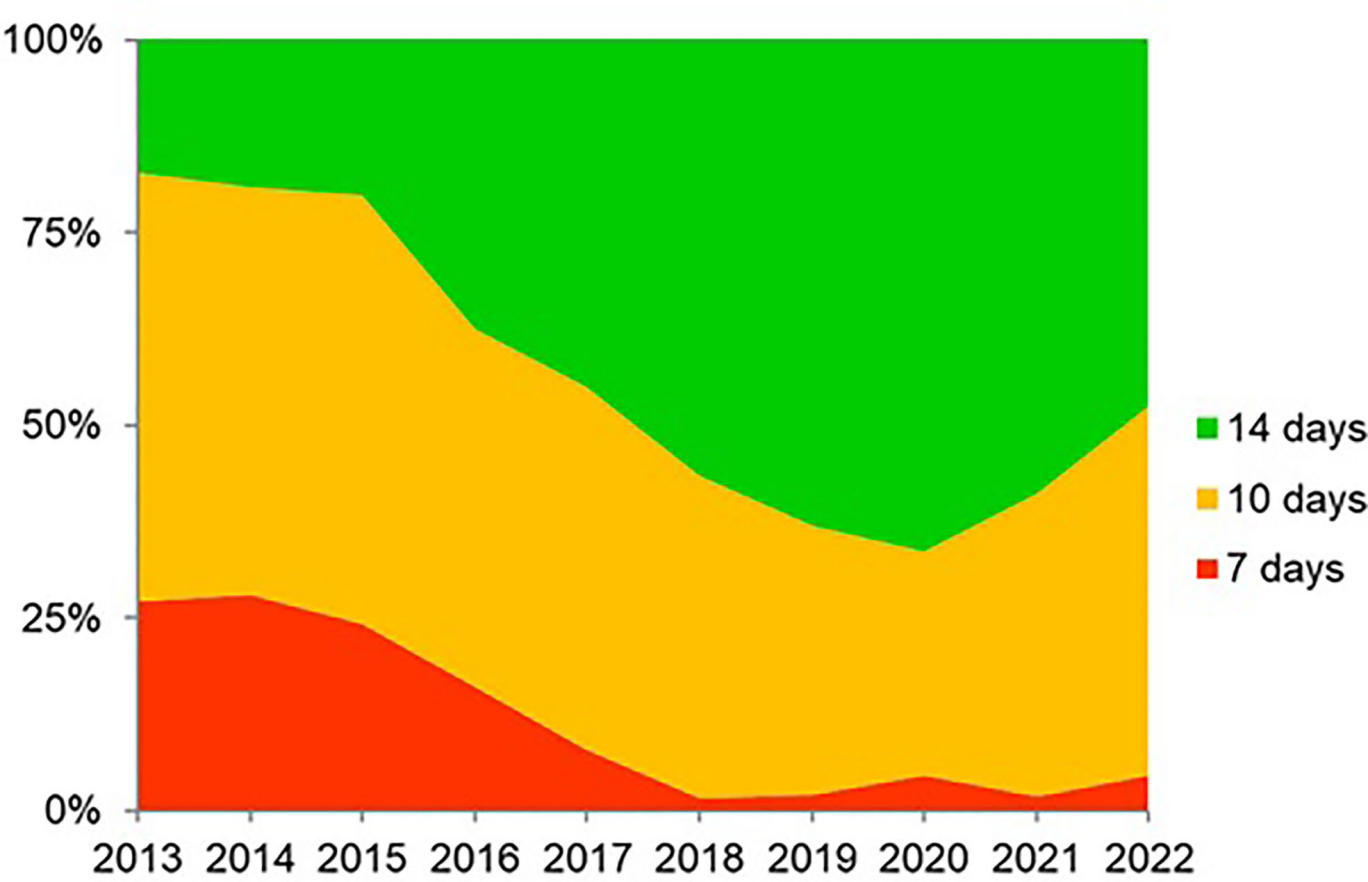
Trends (2013–2022) of 7-, 10-, and 14-day therapy duration (updated in April 2022).

**Figure 5:**
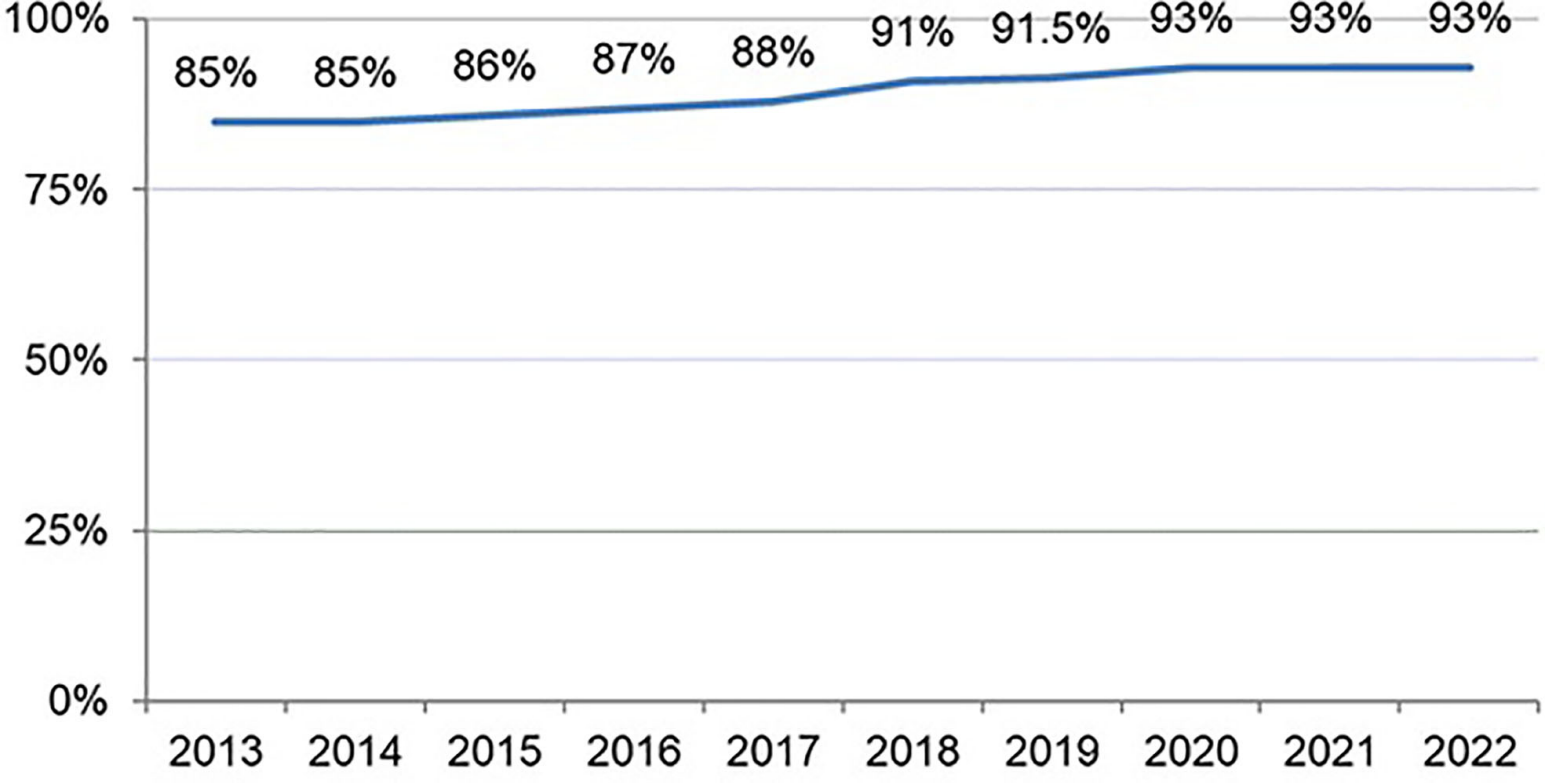
Evolution of first-line treatment by modified intention-to-treat effectiveness from 2013 to 2022 (updated in April 2022).

In total, 21,533 patients from 27 countries were evaluated. Triple therapies were mostly used in southeastern and northern Europe (82%–88%), whereas quadruple therapies were preferred in southwestern and central Europe (63%–82%). Time-trend analysis showed a region-dependent shift in prescriptions: triple therapies were almost abandoned in southwestern and central Europe while still remaining in the east, southeast, and north Europe, areas with low clarithromycin resistance. Sequential and concomitant therapy also did decrease over years but bismuth quadruple therapies increased up to 20% in 2018. As part of the present review, we have updated these figure trends as can be shown in **Figure 2**, updated in April 2022. Over 90% eradication was only obtained with 10-day bismuth quadruple therapies or 14-day concomitant treatment. Also, a shift to longer treatment duration (14 days regimens), as shown in **Figure 3** (with figures updated until April 2022), and higher acid inhibition (high-dose PPIs) trends, as shown in **Figure 4** (with figures also updated in April 2022), were associated with a progressive increase in first-line treatment effectiveness. The shift in prescription trends was associated with an overall effectiveness increase from 84% to 90% in 2018. Moreover, since then, an overall 10% increase in the mITT effectiveness of *H. pylori* first-line empirical eradication treatments has been reported, as is shown in **Figure 5**, updated in April 2022, where overall effectiveness has definitely surpassed the optimal 90% desired threshold previously mentioned.

The results of this study allowed not only continuous assessment on the integration of clinical recommendations agreed on medical consensus, but also the monitoring of the temporal trends of management options and outcomes. These evaluations were aimed to decide on the best possible treatment strategies for improvement (globally and locally), ensuring that routine clinical practice is aligned with best standards of care.

In conclusion, this study indicated that the management of *H. pylori* infection by European gastroenterologists is heterogeneous, frequently suboptimal, and discrepant with current recommendations. Only quadruple therapies lasting at least 10 days were able to achieve over 90% eradication rates. European recommendations are being slowly and heterogeneously incorporated into routine clinical practice, which is associated with a corresponding increase in effectiveness.

#### Combination of bismuth and standard triple therapy eradicates *Helicobacter pylori infection in more than 90% of patients*

The classic bismuth-containing quadruple therapy (PPI, bismuth, tetracycline, and metronidazole) has been recommended as a first-line option in areas with high clarithromycin resistance; however, tetracycline is not available in many countries. Additionally, sequential or concomitant regimens with a PPI, amoxicillin, clarithromycin, and a nitroimidazole have reported better cure rates than standard triple therapy, especially in those areas with moderate to high clarithromycin resistance, but their effectiveness is reduced by dual metronidazole–clarithromycin resistance. Thus, combining bismuth and clarithromycin in the same regimen has been suggested as an alternative option given resistance is not developed to bismuth and that it has an additive or synergistic effect with several antibiotics evading the aforementioned dual bacterial resistance.

The objective of this study was to assess the effectiveness and safety of the combination of bismuth and the standard, clarithromycin-containing triple therapy in the eradication of *H. pylori* infection, using the data from the Hp-EuReg (25). Data were extracted from 2013 up to December 2017.

In total, 1,141 patients (from three countries: 662 from Spain, 402 from Russia, and 77 from Ukraine) receiving empirical bismuth plus standard triple therapy were analyzed. This therapeutic combination was given for 10 days in 321 cases (28%) and for 14 days in 820 cases (72%). The effectiveness of 14-day treatments increased up to 93%. In the multivariate analysis, eradication success was significantly associated with treatment compliance [odds ratio (OR) = 13.0; 95% CI, 5.3–32], a double dose (equivalent to 40 mg of omeprazole) of PPI (OR = 4.7; 95% CI, 1.8–12), and 14-day duration of treatment (OR = 2.0; 95% CI, 1.3–3.2).

In conclusion, the addition of bismuth to 14-day standard triple therapy with clarithromycin and amoxicillin eradicates *H. pylori* infection in more than 90% of patients, resulting in a potential therapeutic gain (10%–20%) in populations with moderate to high clarithromycin resistance, with an acceptable safety profile and level of adherence.

#### European Registry on *Helicobacter pylori* management: Single-capsule bismuth quadruple therapy is effective in real-world clinical practice

Classical bismuth-containing quadruple therapy involves a combination of PPI, metronidazole, and tetracycline, together with bismuth, and has been endorsed in areas with high *H. pylori* resistance to clarithromycin. However, the limited availability of bismuth salts and tetracycline in some countries restricted its use. The appearance of the three‐in‐one single‐capsule bismuth quadruple therapy, containing bismuth, metronidazole, and tetracycline (marketed as Pylera^®^ and registered in some European countries from 2011), prompted the resurgence in the use of bismuth quadruple therapy.

The objective of this study was to evaluate the effectiveness and safety of the single-capsule bismuth quadruple therapy (32). Data were registered from 2013 to January 2020.

Overall, 2,100 patients were prescribed single-capsule bismuth quadruple therapy following the technical sheet (i.e., three capsules every 6 h for 10 days). Most of them (64%) were naïve to treatment. Eradication was over 90% in first-line treatment (95% mITT), and this was maintained as a rescue therapy, both in second-line therapy (89%) and in subsequent lines of therapy (third to sixth line: 92%). Compliance was reported good in all treatment lines, and was the factor most closely associated with the mITT cure rate (OR = 16.0; 95% CI, 7.85–32.5); moreover, high doses of PPI were associated with therapy success, although in a lesser extent (OR = 1.80, 95% CI, 1.14–2.78). The incidence rate of a minimum of one AE was reported by 29% of cases, all mild to moderate in intensity.

In conclusion, in Europe, the single-capsule bismuth quadruple therapy achieved *H. pylori* eradication in approximately 90% of patients in real-world clinical practice, both as a first-line and rescue treatment, with acceptable compliance and safety profile.

#### *Helicobacter pylori* second-line rescue therapy with levofloxacin- and bismuth-containing quadruple therapy, after failure of standard triple or non-bismuth quadruple treatments

As previously mentioned, a rescue regimen comprising a quadruple combination of a PPI, bismuth, tetracycline, and metronidazole has been used as the optimal second-line approach based on the relatively good results reported. However, the complexity of this regimen as well as the incidence of AEs together with the unavailability of tetracycline represent altogether a prescription hurdle. A suggested alternative second-line treatment option is the levofloxacin-containing therapy, ideally combined with bismuth.

The objective of this study was to evaluate the efficacy and tolerability of a second-line quadruple regimen containing levofloxacin and bismuth prescribed for 14 days in those patients whose previous *H. pylori* eradication standard triple therapy (PPI–clarithromycin–amoxicillin) or a non-bismuth quadruple therapy (PPI–clarithromycin–amoxicillin–metronidazole, either sequential or concomitant) had failed (18).

In total, 200 patients from Spain and Italy were evaluated: previous failed therapy included standard clarithromycin triple therapy (131 patients), sequential (32), and concomitant (37). Compliance was reported in 96% of patients. Effectiveness was reported as 90% by mITT and 91.1% in the PP analysis. Cure rates were similar when compared depending on the country (Spain 89.1% vs. Italy 91.9%), the diagnosis (peptic ulcer 96% vs. functional/uninvestigated dyspepsia 89%), and previous treatment (standard triple therapy 88.5% vs. sequential 93.8% vs. concomitant 91.9%). In the multivariate analysis, none of the studied variables were associated with eradication success. AEs were reported in 46% (95% CI, 39–54%) of cases, most commonly nausea (17%), diarrhea (16%), abdominal pain (15%), metallic taste (15%), asthenia (9%), and vomiting (6%). In six cases (3%), AEs were intense but none was classified as serious.

In conclusion, 14-day bismuth- and levofloxacin-containing quadruple therapy is an effective (≥90% cure rate), simple, and safe second-line strategy in patients whose previous standard triple or non-bismuth quadruple therapies have failed.

#### Empirical second-line therapy in 5,000 patients of the European Registry on *Helicobacter pylori* management (Hp-EuReg)

A major reason for treatment failure is acquired *H. pylori* resistance, where those strains surviving an eradication attempt become less susceptible to subsequent therapy. Thus, the ideal choice of a rescue treatment would be guided by previous susceptibility testing. Culture or molecular testing may not be available in routine clinical practice and, therefore, empiric treatment needs to be optimized (44).

The objective of this study was to assess the effectiveness and safety of empirical second-line treatment in Europe (33). All *H. pylori-*infected adult patients with a previous treatment eradication attempt were data extracted from 2013 to February 2021.

**Figure 6:**
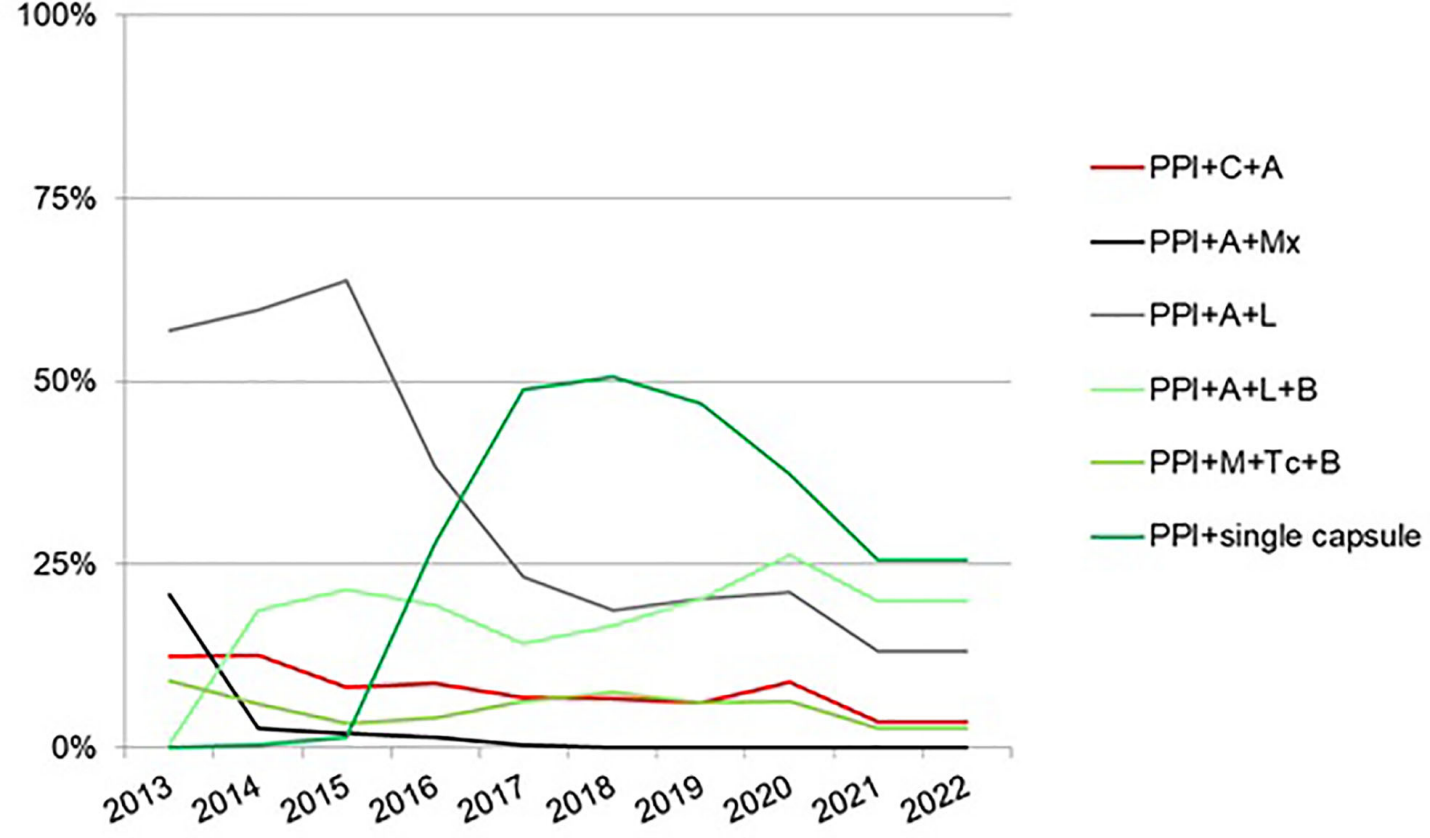
Time trends in the most common second-line empirical treatment prescriptions from 2013 to 2022 (updated in April 2022). A—amoxicillin; B—bismuth; C—clarithromycin; L—levofloxacin; M—metronidazole; Mx—moxifloxacin; Tc—tetracycline; single capsule—as the bismuth quadruple therapy containing metronidazole, tetracycline, and bismuth.

**Figure 7:**
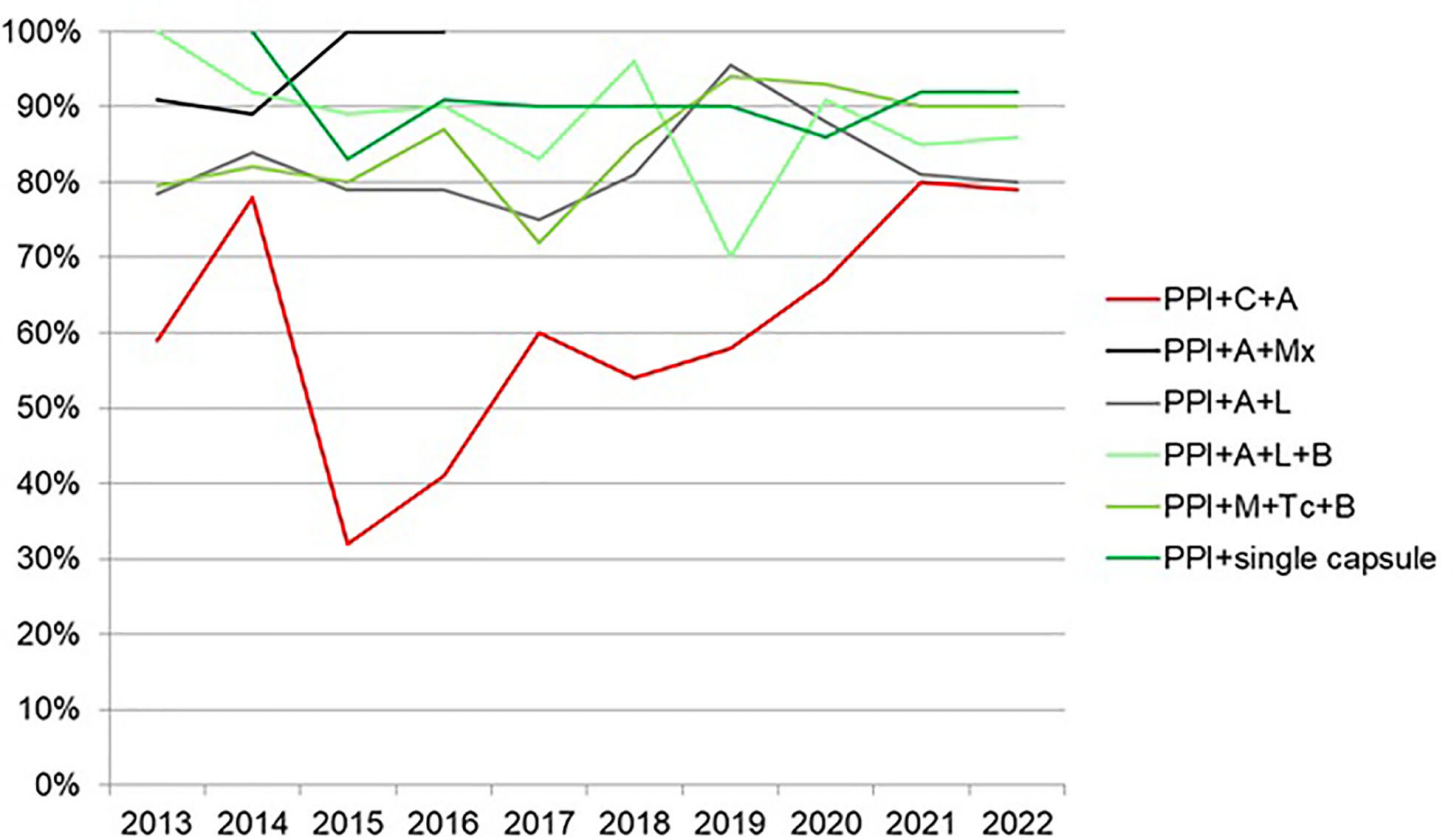
Trends in the effectiveness by modified intention-to treat of the most common second-line empirical treatment from 2013 to 2022 (updated in April 2022). A—amoxicillin; B—bismuth; C—clarithromycin; L—levofloxacin; M—metronidazole; Mx—moxifloxacin; Tc—tetracycline; single capsule—as the bismuth quadruple therapy containing metronidazole, tetracycline, and bismuth.

In total, 5,055 cases from 27 countries received second-line empirical treatment. Overall, 87 different second-line treatments were registered. Triple therapy with amoxicillin and levofloxacin was prescribed most commonly (33%), followed by bismuth quadruple therapy (single-capsule, 17%), and levofloxacin-bismuth quadruple therapy (13%), as shown in **Figure 6**, updated in April 2022. The overall effectiveness of empirical second-line therapy was reported as 84% (95% CI, 82–84%) by mITT. After the failure of first-line clarithromycin-containing treatment, optimal eradication was obtained with moxifloxacin-containing triple therapy (91%) or levofloxacin-bismuth quadruple therapy (89%). In patients receiving triple therapy containing levofloxacin or moxifloxacin, and levofloxacin-bismuth quadruple treatment, cure rates were optimized with 14-day regimens using high doses of PPIs. However, three-in-one single-capsule or levofloxacin-bismuth quadruple therapy produced reliable eradication rates regardless of PPI dose, duration of therapy, or previous first-line treatment. Effectiveness of those most commonly prescribed second-line empirical treatments is reported in **Figure 7**, updated in April 2022. The overall incidence of AEs was 28%, and most (85%) were mild.

In conclusion, second-line empirical regimens including 14-day quinolone triple therapies, 14-day levofloxacin–bismuth quadruple therapy, 14-day tetracycline–bismuth classic quadruple therapy, and 10-day bismuth quadruple therapy (as a single-capsule) provided optimal effectiveness. However, many other second-line treatments evaluated reported low eradication rates.

#### *Helicobacter pylori* first-line and rescue treatments in patients allergic to penicillin: Experience from the European Registry on *H. pylori* management (Hp-EuReg)

Experience of *H. pylori* management in patients allergic to penicillin is very scarce. First-line treatment often includes a triple therapy with a PPI, clarithromycin, and metronidazole. However, more recently, a quadruple therapy including a PPI, bismuth, tetracycline, and metronidazole has been recommended.

The objective of this study was to assess the effectiveness and tolerability of first-line and rescue treatments in those cases that were allergic to penicillin (26). Patients with penicillin allergy were data extracted from 2013 to June 2019.

In total, 1,084 patients were analyzed. In those treatment-naïve cases, the effectiveness of the regimens that were most frequently administered was 69% with PPI–clarithromycin–metronidazole, and 91% with PPI–tetracycline–metronidazole–bismuth, with significant differences between treatment groups. In second-line treatment, after the failure of a triple therapy with PPI–clarithromycin–metronidazole, two rescue options showed similar cure rates: bismuth quadruple therapy with PPI–tetracycline–metronidazole–bismuth (78%) and triple therapy with PPI–clarithromycin–levofloxacin (71%), with no differences between groups. In third-line treatment, after the failure of PPI–clarithromycin–metronidazole and PPI–clarithromycin–levofloxacin, the bismuth quadruple therapy with PPI–tetracycline–metronidazole-bismuth was successful in 75% of cases.

In conclusion, in patients allergic to penicillin, a triple combination with PPI, clarithromycin, and metronidazole should not be generally recommended as a first-line treatment, while a quadruple regimen with PPI, tetracycline, metronidazole, and bismuth seems to be a better option.

#### Adverse event profile during the treatment of Helicobacter pylori: A real-world experience of 22,000 patients from the European Registry on *H. pylori* management (Hp-EuReg)

The safety of *H. pylori* eradication treatments and to what extent AEs influence therapeutic compliance in clinical practice are hardly known.

The objective of this study was to evaluate the incidence rate, type, intensity, and duration of AEs, and their effect on treatment adherence, the most frequent prescriptions in Europe (34). All eradication treatments and their corresponding safety profile were collected. AEs were graded depending on the patient-reported intensity of symptoms as mild, moderate, or severe, and as serious AEs.

In total, 22,492 patients were registered and different treatments prescribed caused at least one AE in 23% of the patients; there were nine kinds of different AEs, the most recurrent of which were as follows: taste disturbance (7%), diarrhea (7%), nausea (6%), and abdominal pain (3%), where the majority (57%) were mild. The average duration of AEs was 7.3 days, ranging from 1 to 45 days. On the other hand, the incidence of AEs was 22% in the 14 most frequently prescribed therapies assessed (4,298 cases): the classic bismuth-based quadruple therapy with metronidazole and either tetracycline or doxycycline was the worst tolerated (37% and 33%, respectively), followed by the bismuth-containing quadruple together with amoxicillin and clarithromycin, josamycin, or levofloxacin, (34%, 32%, and 32%, respectively). The greater proportion of AEs was reported as mild (57%), 6% severe, and only 0.08% serious. The treatment compliance rate was 97%, and only 1.3% of the patients discontinued treatment due to AEs. Longer treatment durations were significantly associated with a higher incidence of AEs in standard triple, concomitant, bismuth quadruple, and levofloxacin triple or quadruple therapies. The incidence of AEs for each most frequent treatment is included in **Table 3** updated in April 2022.

**Table 3:** Adverse events of most frequent *H. pylori* eradication treatments in Europe (updated in April 2022).

Regimen	Number of patients with adverse events	%	95% CI
**Quadruple-PPI+M+Tc+B**	285	32	(29–35)
**Quadruple-PPI+M+D+B**	69	32	(26–38)
**Triple-PPI+A+M**	371	31	(28–33)
**Quadruple-PPI+C+A+B**	1,429	30	(28–31)
**Quadruple-PPI+C+A+M**	1,909	29	(28–30)
**Single-capsule+PPI**	1,823	28	(27–29)
**Sequential-PPI+C+A+M**	193	27	(23–30)
**Quadruple-PPI+C+A+T**	111	26.5	(22–31)
**Quadruple-PPI+A+L+B**	250	26	(23–29)
**Triple-PPI+A+L**	568	21	(19–23)
**Triple-PPI+C+A**	2,480	19	(18–19)
**Triple-PPI+C+M**	252	16	(14–17)
**Total**	9,740	24.5	(24–25)

95% CI—95% confidence interval; PPI—proton pump inhibitor; A—amoxicillin; B—bismuth salts; C—clarithromycin; L—levofloxacin; M—metronidazole; T—tinidazole; Tc—tetracycline; single capsule—marketed as Pylera^®^ containing bismuth, tetracycline, and metronidazole.

In conclusion, *H. pylori* eradication treatment tends to be safe in real clinical practice. Treatments caused at least one AE in 1/4 of the cases. The highest proportion of AEs was reported as mild, and only <1% of AEs were serious. The presence of AEs does not significantly affect treatment adherence.

#### The role of statins on Helicobacter pylori eradication: Results from the European Registry on *H. pylori* management (Hp-EuReg)

In order to enhance *H. pylori* treatment success, several optimization strategies have been suggested, such as, for instance, extending therapy length, increasing the potency of the acid inhibition used, adding bismuth, or adding more antibiotics to the regimen prescribed. In this context, co-treatment with statins, linked to their role in the cholesterol synthesis cascade, was thought to have a synergistic effect in the healing of gastric inflammation as well as to increase the eradication rate against *H. pylori* infection (45).

The objective of this study was to assess whether the use of statins prescribed for cardiovascular prevention and chronically used, and prescribed concomitantly with an *H. pylori* eradication therapy could modify the effectiveness rates of eradication treatments with regard to (a) the type of eradication therapy, in both the empirical and culture-guided groups, or (b) the statins prescribed, and ultimately whether these statins would modify the AE rates (35). In order to analyze data, patients were further divided into two groups: the ones receiving statins during the eradication therapy and the non-statin consumers. Data were collected from 2013 to August 2021.

Overall, 9,988 and 705 patients received empirical and culture-guided treatment, respectively. The type of statin was reported in 13% of all statin consumers: simvastatin (*n* = 155, 45%), atorvastatin (*n* = 134, 39%), rosuvastatin (*n* = 37, 11%), and a last group named “others” (*n* = 17, 5%) including other statins (e.g., pravastatin, pitavastatin, lovastatin, or fluvastatin). Overall, the statin use was associated with higher effectiveness in the empirical group (OR = 1.3; 95% CI, 1.1–1.5), but no association was found with first-line treatment effectiveness (*n* = 7,738); however, as an exception, statin use was specifically associated with lower effectiveness of empirical standard triple therapy (OR = 0.76; 95%CI, 0.59–0.99). In the rescue (second to sixth line) empirical therapy group (*n* = 2,228), statin consumers were associated with higher effectiveness (OR = 1.9; 95% CI, 1.4–2.6). However, in this same rescue group, the single-capsule bismuth quadruple therapy, when prescribed together with statins, was the only treatment scheme significantly associated with higher effectiveness (OR = 2.8; 95% CI, 1.3–5.7). Additionally, the specific analysis according to the different statins used (simvastatin, atorvastatin, rosuvastatin, and other statins) showed no association between any of them and the effectiveness or safety of the eradication therapies, either globally or in the sub-analyses of the most frequently used regimens.

In conclusion, no consistent association was found between statin use and *H. pylori* therapy effectiveness. Therefore, the addition of statins to the usual *H. pylori* treatment cannot be currently recommended to improve cure rates.

#### Antibiotic resistance prevalence and trends in patients infected with Helicobacter pylori in the period 2013–2020: Results of the European Registry on *H. pylori* management (Hp-EuReg)

Bacterial antibiotic resistance changes over time depending on multiple factors (46); therefore, it is essential to monitor the susceptibility trends to reduce the resistance impact on the effectiveness of various treatments.

The objective of this study was to conduct a time-trend analysis of *H. pylori* primary resistance to antibiotics in Europe (36). Therefore, all infected adult patients diagnosed with culture and antimicrobial susceptibility testing with a positive result were included in the analysis. Data were collected from 2013 to December 2020.

**Figure 8:**
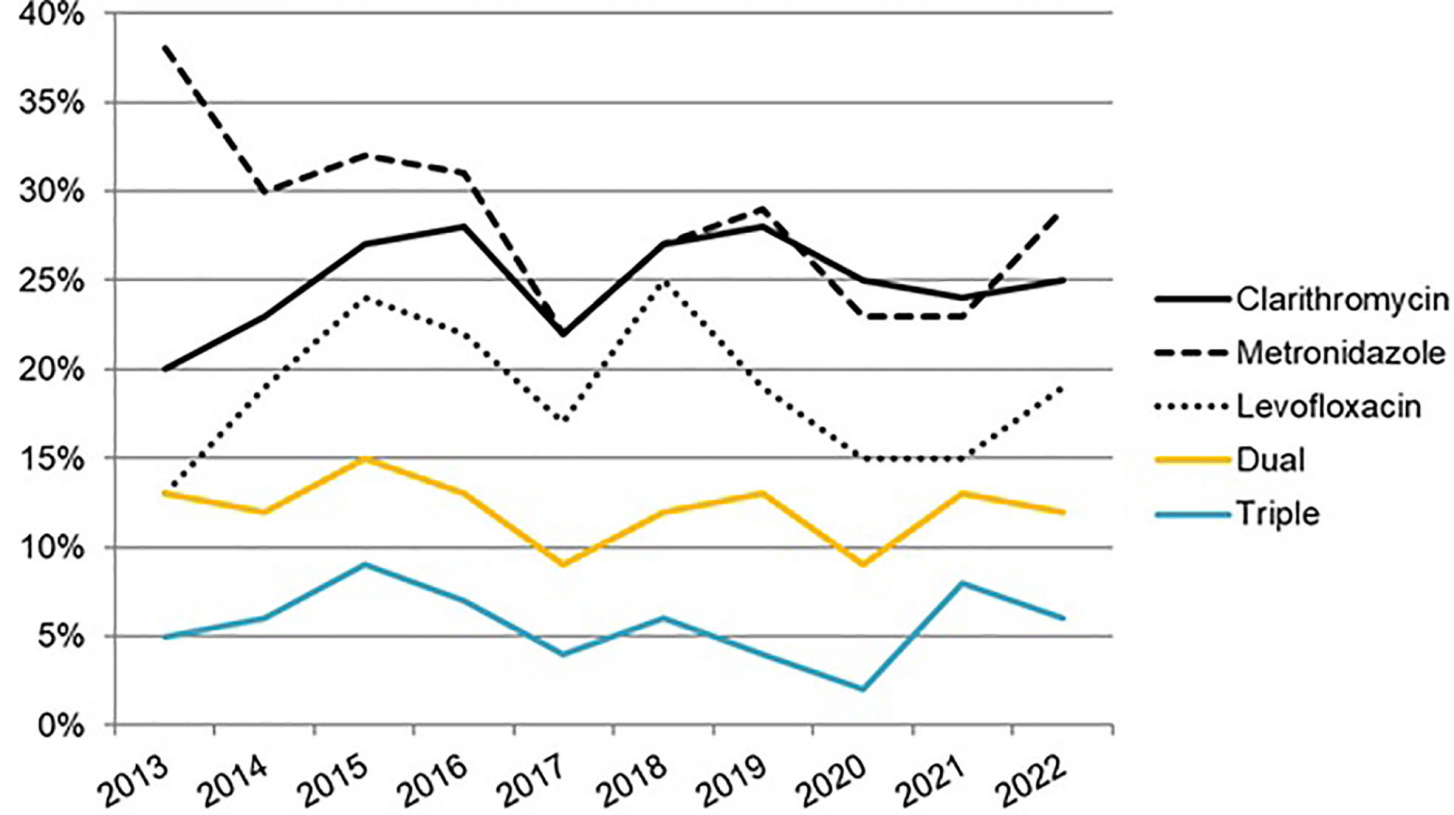
Evolution of *H. pylori* antibiotic resistance in naïve patients from 2013 to 2022 (updated in April 2022). Dual resistance—to both clarithromycin and metronidazole (regardless of any other antibiotic resistance); Triple resistance—to clarithromycin, metronidazole and levofloxacin (regardless of any other antibiotic resistance).

In total, 2,852 (7% of the total sample size) treatment-naïve cases with culture and antimicrobial susceptibility testing were included for analysis. The country-by-country allocation of cases was as follows: 2,360 (59%) in Italy; 454 (11.4%) in Spain; 368 (9.3%) in Norway; 248 (6.2%) in Greece; 211 (5.3%) in Slovenia; 110 (2.8%) in Israel; 45 (1.1%) in France; and 40 (1%) in Ireland. The amount of positive cultures was reduced by 35% between the period 2013–2016 and 2017–2020. No bacterial antibiotic resistance was reported in 48% of the patients, and frequently those detected *H. pylori* antibiotic resistances resulted in the following: metronidazole (30%), clarithromycin (25%), and levofloxacin (20%), whereas resistances to tetracycline and amoxicillin were below 1%. Dual clarithromycin–metronidazole, and triple clarithromycin–metronidazole–levofloxacin resistances were reported by 13% and 6% of the cases, respectively. The average resistance rate to clarithromycin was 25% (95% CI, 16–34%), which was highest in 2016, with 34% of cases. Levofloxacin resistance persisted on average above 15%. Dual resistance to both clarithromycin and metronidazole antibiotics was higher than 10% throughout most of the period studied, and triple resistance to all three clarithromycin, metronidazole, and levofloxacin antibiotics was reported to be over 5%. When the 2013–2016 period was compared to 2017–2020, all antibiotics’ primary resistance rates showed a decreasing trend, but a greater significant decrease was observed in the metronidazole resistance rate between the same periods (33% and 24%, *p* < 0.001; respectively), as it is likewise reported in **Figure 8**, updated in April 2022, where the evolution of all antibiotics’ prevalence is reported and where the metronidazole prevalence is currently still decreasing. Also, the prevalence of *H. pylori* resistance in south Europe was greater than in the north (Norway) (56% vs. 31.5%, respectively; *p* < 0.005). In patients prescribed second or subsequent lines of treatment, bacterial antibiotic resistance was over 80%: following a first eradication treatment attempt, the clarithromycin bacterial resistance was over 60% and that of levofloxacin was 28%, reaching over 45% in the latter case, when the patient had received more than two eradication therapies. After the failure of the first eradication treatment, dual and triple resistances were found in 43% and 19% of cases, respectively. These resistances gradually augmented after the failure of four eradication therapies, providing rates of 63% and 39%, respectively.

In conclusion, culture and antimicrobial susceptibility testing for *H. pylori* are scarcely performed (<10%) in Europe. In naïve patients, *H. pylori* resistance to clarithromycin remained above 15% throughout the period 2013–2020, and resistance to levofloxacin, as well as dual or triple resistances, was high. A progressive decrease in metronidazole resistance is observed.

#### Experience with rifabutin-containing therapy in 500 patients from the European Registry on *Helicobacter pylori* management (Hp-EuReg)

First-line anti-*H. pylori* eradication treatments have been quite properly evaluated; however, identifying the best treatment option in those patients needing a rescue therapy is still necessary. Rifabutin is a recognized antimicrobial agent belonging to the group of the S-rifamycin derivatives and has been successfully used previously, among others, for the treatment of atypical *Mycobacterium* infections. It has been described that *H. pylori* has high *in vitro* sensitivity to rifabutin, and so, this antibiotic might be effective against the infection as it does not share the same resistance mechanisms as other usual antibiotics such as clarithromycin, metronidazole, or levofloxacin, which are commonly used in eradication regimens.

The objective of this study was to evaluate the effectiveness and safety of those rifabutin-containing regimens against *H. pylori *(40). Thus, all rifabutin-treated cases were recorded between 2013 and 2021, in the e-CRF of AEG-REDCap.

Overall, 500 patients from seven countries were treated with a total of 18 different rifabutin-containing regimens. The majority of cases (90% of the data) were encompassed within the three following countries: Italy (333 patients) and Spain (117 patients) followed by Israel (33 patients). A result of culture testing was reported in 63% of patients: dual clarithromycin–metronidazole resistance was 46%, and triple clarithromycin, metronidazole, and levofloxacin resistance was 39%. Rifabutin was prescribed in 87% of patients as part of a triple therapy together with a PPI and amoxicillin, and in further 6% of the cases, bismuth was combined with the aforementioned triple regimen. Rifabutin was administered as 150 mg once a day (56%) or 150 mg twice a day, i.e., 300 mg daily (41%). The therapies were frequently combined with low-dose (46%) or high-dose PPIs (46%), and prescribed most commonly for 12 days (58%). Rifabutin was mostly prescribed in second-line (32%), third-line (25%), and fourth-line (27%) treatment, providing overall 78%, 80%, and 66% mITT eradication rates, respectively. Overall effectiveness with rifabutin at 150 mg once a day was reported to be higher (78%) as compared to 300 mg/day (67%), although differences were not statistically significant. The triple therapy with amoxicillin and rifabutin provided an overall effectiveness of 73% (*n* = 265/363); however, in second- and third-line treatment (77%, *n* = 103/133 and 79%, *n* = 66/84, respectively), eradication rates were reported to be higher than in fourth-line treatment (64%, *n* = 53/83), with no significant effectiveness differences among treatment lines. No significant differences were found between treatment durations; however, when a high-dose PPI was used, better outcomes were provided with this same regimen (87.5%) than when low (66%) or standard PPI doses (53%) were used, showing statistically significant differences in the cure rate (*p* < 0.001). In fourth-line treatment, the overall effectiveness achieved with quadruple therapy containing a PPI, amoxicillin, rifabutin, and bismuth was 68% (*n* = 21/31). Overall treatment compliance was 89%. The incidence rate of at least one AE was reported by 26% of the cases (most often nausea), and only one serious AE (0.2%) was registered in a case with leukopenia and thrombocytopenia with fever requiring hospitalization.

In conclusion, those rifabutin-containing regimens are an effective and safe treatment approach after one or even several *H. pylori* eradication treatment failures.

#### Effectiveness and safety of high-dose dual therapy: Results of the European Registry on *H. pylori management (Hp-EuReg)*

Several randomized clinical trials and meta-analyses, mostly from Asian countries, have reported optimal efficacy and safety with high doses of amoxicillin and a PPI—that is, high-dose dual therapy (HDDT)—when prescribed either as first-line or as a rescue treatment.

The objective of this study was to evaluate the frequency of use, the effectiveness, the compliance, and the safety of the HDDT in Europe (43).

In this study, 60 patients were prescribed HDDT: 19 cases were treatment-naïve and 41 were treated as rescue therapy. Overall, HDDT effectiveness was 52% (per-protocol) and 51% (by modified intention-to-treat) with no statistical differences between treatment lines. Effectiveness decreased in those who had been previously treated with metronidazole, tetracycline, or rifabutin. The addition of bismuth to HDDT did not increase the cure rates. Safety was reported as a 30% incidence rate of at least one AE (diarrhea was the most common in 20% of the cases). No serious AEs were registered.

In conclusion, HDDT (with or without bismuth) does not represent a good therapeutic option in Europe in any line of treatment.

#### Room for improvement in the treatment of Helicobacter pylori infection: Lessons from the European Registry on *H. pylori* management (Hp-EuReg)

The management of the infection of *H. pylori* involves continuous decision-making, and each choice is open to probable mistakes.

The objective of this study was to assess those common mistakes in the *H. pylori* eradication treatment (41). For this purpose, countries recruiting more than 1,000 patients, with data collected up to 2019, were included in the analysis.

In total, 26,340 patients were evaluated among the highest recruiters, hereby listed by descending order: Spain (14,751 cases), Russia (4,462 cases), Italy (3,289 cases), Slovenia (3,193 cases), and Lithuania (1,226 cases), representing 80% of the registry up until the data extraction date. The most common mistakes (percentages) found were as follows: (1) using the standard triple therapy where it is ineffective (46%); (2) prescribing eradication therapy for only 7 to 10 days (69%); (3) using a low dose of PPIs (48%); (4) in patients allergic to penicillin, always prescribing a triple therapy with clarithromycin and metronidazole (38%); (5) repeating certain antibiotics after eradication failure (>15%); (6) failing to consider the importance of compliance with treatment (2%); and, finally, (7) not checking the eradication success (6%). All these mistakes have been reviewed at the time of writing the present manuscript, and the list with the updated figures can be checked in **Table 4**, updated in April 2022. The analysis of the evolution of treatment management showed gradual greater adherence to current clinical guidelines.

**Table 4:** Most common mistakes in the management of *H. pylori* treatment in Europe (updated in April 2022).

Common mistake	Error
1. **To use the standard triple therapy where it is ineffective**	**44%**
2. **To prescribe eradication therapy for only 7 to 10 days**	**61%**
3. **To use a low dose of proton pump inhibitors**	**43%**
4. **In patients allergic to penicillin, to prescribe always a triple therapy with clarithromycin and metronidazole**	**32%**
5. **To repeat certain antibiotics after eradication failure**	**>16%**
6. **Failing to consider the importance of compliance with treatment**	**2%**
7. **Not to check the eradication success**	**2%**

In conclusion, the management of the infection of *H. pylori* by many European gastroenterologists is heterogeneous, often suboptimal and contradictory with current recommendations. Clinical practice is continuously adjusting to the most up-to-date recommendations, even though this change is delayed and slow.

### Local data, by country

To date, 14 studies have been published locally, providing a “country experience”. Overall, three studies evaluated data in Spain (19, 27, 28), one in Greece (42), two in Slovenia (22, 37), one in Lithuania (38), one in Israel (39), five in Russia (20, 21, 23, 29, 30), and one in Hungary (24). A synthesis of the most relevant conclusions from these studies is also reported in **Table 2**.

Additionally, the results on two of the Spanish studies have been further detailed below as data could potentially be extrapolated to other European countries, given the large sample size evaluated.

One of the Spanish studies (27) evaluated the effectiveness of first- and second-line *H. pylori* treatment in Spain, where the empirical prescription is generally recommended. The analysis was performed with data extracted from 2013 to June 2019.

In total, the evaluation of 53 Spanish hospitals was performed, with 10,267 patients receiving a first-line empirical treatment. The best first-line effectiveness outcomes were obtained with the 10-day single-capsule bismuth quadruple therapy (95%) and with the 14-day non-bismuth quadruple concomitant therapies (PPI–bismuth–clarithromycin–amoxicillin, 91%; and PPI–clarithromycin–amoxicillin–metronidazole, 92%). However, the second most frequently prescribed first-line treatment (after the non-bismuth quadruple concomitant therapy), that is, the standard triple therapy with amoxicillin and clarithromycin, provided only 83% effectiveness. The overall first-line eradication rate of triple therapies was below 75%. Second-line therapy was prescribed to 2,448 patients, the most effective of which were the 14-day triple quinolone (PPI–amoxicillin–levofloxacin/moxifloxacin, 92% and 89%, respectively), the 14-day bismuth–levofloxacin quadruple schemes (PPI–bismuth–levofloxacin–amoxicillin, 90%), and the 10-day bismuth single capsule (88.5%). Second-line triple regimens provided likewise overall suboptimal eradication rates (<85%). Compliance, longer duration, and higher acid inhibition were associated with higher mITT effectiveness. Thus, optimization (longer treatment duration, i.e., > 14 days, and high-dose PPIs) achieved over 90% eradication rate.

In conclusion, in Spain, standard triple therapy with amoxicillin and clarithromycin prescribed empirically should be abandoned. In first-line therapy, bismuth-containing quadruple therapy for 10 days (single capsule) or for 14 days (concomitant and bismuth–clarithromycin quadruple therapies regimens) obtained the best effectiveness results. In second-line therapy, bismuth-containing quadruple therapy for 10 days (single capsule) and 14-day quinolone-containing therapies, either with or without bismuth, provided the highest cure rates.

Another important study evaluating a Spanish cohort (28) assessed the effectiveness and safety in the eradication of *H. pylori* of third-line quadruple therapies containing bismuth salts, metronidazole, and either tetracycline (standard form or with the three-in-one single capsule) or doxycycline. Thus, three bismuth quadruple regimens were analyzed in this study: traditional bismuth quadruple with a PPI, bismuth salts (120 mg/6 h or 240 mg/12 h), metronidazole (500 mg/8 h), and tetracycline (500 mg/6 h) (BQT-Tet); the same treatment substituting tetracycline with doxycycline 100 mg/12 h (BQT-Dox); and the three-in-one single-capsule commercial version (BQT-three-in-one; Pylera^®^). Data were extracted from 2013 to December 2019.

In total, 443 Spanish cases received one of the three regimens and were analyzed. Most of these patients had received triple therapy with clarithromycin in first-line treatment (86%) and with levofloxacin in second-line treatment (73%). The remaining received quadruple therapy. Third-line treatments were prescribed in 10- and 14-day regimens. Highest effectiveness was found in those taking 10-day bismuth quadruple therapy three-in-one single-capsule (88%; 95% CI, 83–92) or 14-day bismuth quadruple therapy with tetracycline (82%; 95% CI, 71–93%). Effectiveness with quadruple treatment with doxycycline was below 70%, regardless of the length of treatment or the PPI dose.

In conclusion, third-line *H. pylori* eradication with bismuth quadruple treatment (after failure with clarithromycin and levofloxacin) offers acceptable effectiveness and safety. Doxycycline seems to be less effective and therefore should not be recommended.

## Final reflections

The Hp-EuReg surpasses the procedure described in the protocol; it has been proven as a new approach to evidence‐based, where, through a systematic auditing process, accurate global and locally applicable recommendations can be performed thanks to both the trial evidence and practitioner experience gathered from several perspectives such as medical, scientific, economic, and social perspectives.

The analyses of the various studies derived from the Hp-EuReg cover the patients’ reported outcomes mainly on the diagnosis and eradication treatment strategies, serving to the final implementation and optimization of *H. pylori* clinical management. Therefore, the results from the Hp‐EuReg represent valuable data for present and future consensus conferences, and up-to-date precise evidence to health authorities and medical societies useful in the preparation of policies and actions to benefit the health assistance to their populations. It should also speed up the time for new approaches, e.g., molecular testing before treatment, to become normal practice in clinical settings.

In essence, the Hp-EuReg has an undeniable influence on the routine clinical practice of European gastroenterologists, improving *H. pylori* eradication treatment success, allowing to make recommendations (reinforcing current guidelines) and potentially serving as a model for other diseases.

## Author contributions

OP designed the protocol, performed the review of the included studies, synthesized the data, wrote the manuscript draft, and approved the submitted manuscript. LM, NG-M, AC-C, IP, and FM critically reviewed the manuscript draft and approved the final submitted manuscript. CO’M planned the study, critically reviewed the manuscript draft, and approved the final submitted manuscript. JG designed the protocol, critically reviewed the manuscript drafts, and approved the final submitted manuscript.

## Funding

The Hp-EuReg project was promoted and funded by the European Helicobacter and Microbiota Study Group (EHMSG), the Spanish Association of Gastroenterology (AEG), and the Centro de Investigación Biomédica en Red de Enfermedades Hepáticas y Digestivas (CIBERehd).

## Conflict of interest

JG has served as speaker, consultant, and advisory member for or has received research funding from Mayoly, Allergan, Diasorin, Gebro Pharma, and Richen. OP has received research funding from Mayoly and Allergan.

The remaining authors declare that the research was conducted in the absence of any commercial or financial relationships that could be construed as a potential conflict of interest.

## Publisher’s note

All claims expressed in this article are solely those of the authors and do not necessarily represent those of their affiliated organizations, or those of the publisher, the editors and the reviewers. Any product that may be evaluated in this article, or claim that may be made by its manufacturer, is not guaranteed or endorsed by the publisher.
