# Supplementary material for: European Registry on Helicobacter pylori Management (Hp-EuReg): Most relevant results for clinical practice
Source: Front Gastroenterol (Lausanne). 2022 Aug 17;1:965982. doi: 10.3389/fgstr.2022.965982 (PMC12952441; doi:10.3389/fgstr.2022.965982)
Supplement: Supplementary file 1 [file Table_1.docx]

**Supplementary Table 1. Manuscripts in preparation**

| Title of the study |
| --- |
| 1. Prescriptions, effectiveness and safety of *H. pylori* eradication regimens in the elderly |
| 1. Patterns and relevance of compliance with eradication treatment in Europe |
| 1. Patterns, effectiveness and safety of proton pump inhibitor prescription for *H. pylori* eradication in Europe |
| 1. Patterns, effectiveness and safety of antibiotic prescription for *H. pylori* eradication in Europe |
| 1. Role of bismuth in *H. pylori* eradication in Europe: effectiveness and safety |
| 1. *H. pylori* diagnostic tests used in Europe |
| 1. Role of probiotics in *H. pylori* eradication in Europe: effectiveness and safety |
| 1. Third to sixth-line rescue therapies for *H. pylori* infection in Europe |
| 1. Influence on antibiotic resistance on efficacy *H. pylori* eradication therapy in Europe |
| 1. Using bismuth quadruple three-in-one single capsule thrice-a-day increases the effectiveness versus the usual four times-a-day schedule |
| 1. Clinical phenotyping through machine learning on *H. pylori* treatment-naïve patients in Europe |
| 1. Effectiveness of empirical first-line therapy in Russia from 2013 to 2021 |
| 1. Effectiveness and safety of furazolidone-based *H. pylori* eradication treatments in Russia |
| 1. Effectiveness of first and second-line *H. pylori* empirical treatment in Italy |
| 1. Current trends in the management of *H. pylori* infection in Serbia |
| 1. *H. pylori* treatment prescriptions in Latvia: gastroenterologists versus general practitioners |
| 1. The Azerbaijan history: diagnosis and treatment for *H. pylori* infection |
| 1. Current trends in the management of *H. pylori* infection in Ukraine |
| 1. Prescriptions and effect of *H. pylori* antibiotic resistance on eradication treatments in Norway |
| 1. Patterns and *H. pylori* treatment effectiveness in Croatia |
|  |
